# Supplementary material for: Mechanisms governing the pioneering and redistribution capabilities of the non-classical pioneer PU.1
Source: Nat Commun. 2020 Jan 21;11:402. doi: 10.1038/s41467-019-13960-2 (PMC6972792; doi:10.1038/s41467-019-13960-2)
Supplement: Supplementary file 7 — Source data [file 41467_2019_13960_MOESM7_ESM.zip › Source_Data/Figure2/Figure2F_MetascapeResults/AnalysisReport.html]

## Metascape Gene List Analysis Report

metascape.org^1^

### Enrichment Summary

Figure 1. Heatmap of enriched terms across input gene lists, colored by p-values.

### Gene Lists

User-provided gene identifiers are first converted into their H. sapiens Entrez gene IDs using the latest database (updated on 2018-01-01). If multiple identifiers correspond to the same Entrez gene ID, they will be considered as a single Entrez gene ID in downstream analyses. The gene lists are summarized in Table 1.

Table 1. Statistics of input gene lists.

| Name | Total | Unique |
| --- | --- | --- |
| hits | 283 | 221 |

### Gene Annotation

The following are the list of annotations retrieved from the latest database (updated on 2018-01-01) (Table 2).

Table 2. Gene annotations extracted

| Name | Type | Description |
| --- | --- | --- |
| Gene Symbol | Description | Primary HUGO gene symbol. |
| Description | Description | Short description. |
| Biological Process (GO) | Function/Location | Descriptions summarized based on gene ontology database, where up to three most informative GO terms are kept. |
| Kinase Class (UniProt) | Function/Location | Detailed kinase classes. |
| Protein Function (Protein Atlas) | Function/Location | Protein Function (Protein Atlas) |
| Subcellular Location (Protein Atlas) | Function/Location | Sucellular Location (Protein Atlas) |
| Drug (DrugBank) | Genotype/Phenotype/Disease | Drug information for the given gene as target. |
| Canonical Pathways | Ontology | Canonical Pathways |
| Hallmark Gene Sets | Ontology | Hallmark Gene Sets |

### Pathway and Process Enrichment Analysis

For each given gene list, pathway and process enrichment analysis was carried out with the following ontology sources: KEGG Pathway, GO Biological Processes, Reactome Gene Sets, Canonical Pathways and CORUM. All genes in the genome were used as the enrichment background. Terms with p-value < 0.01, minimum count 3, and enrichment factor > 1.5 (enrichment factor is the ratio between observed count and the count expected by chance) are collected and grouped into clusters based on their membership similarities. More specifically, p-values are calculated based on accumulative hypergeometric distribution^2^, q-values are calculated using the Banjamini-Hochberg procedure to account for multiple testing^3^. Kappa scores^4^ were used as the similarity metric when performing hierachical clustering on the enriched terms and then sub-trees with similarity > 0.3 are considered a cluster. The most statistically significant term within a cluster is chosen as the one representing the cluster.

Table 3. Top 20 clusters with their representative enriched terms (one per cluster). "Count" is the number of genes in the user-provided lists with membership in the given ontology term. "%" is the percentage of total user-provided genes that are found in the given ontology term (only input genes with at least one ontology term annotation are included in the calculation). "Log10(P)" is the p-value in log base 10. "Log10(q)" is the multi-test adjusted p-value in log base 10.

| GO | Category | Description | Count | % | Log10(P) | Log10(q) |
| --- | --- | --- | --- | --- | --- | --- |
| GO:0002274 | GO Biological Processes | myeloid leukocyte activation | 22 | 10.3286384977 | -6.8527132927 | -2.91313534561 |
| GO:0045576 | GO Biological Processes | mast cell activation | 6 | 2.81690140845 | -4.6215556579 | -1.16629738093 |
| GO:0050900 | GO Biological Processes | leukocyte migration | 14 | 6.57276995305 | -3.79587773596 | -0.671612678034 |
| GO:0007264 | GO Biological Processes | small GTPase mediated signal transduction | 14 | 6.57276995305 | -3.70165302584 | -0.63174563024 |
| GO:0042100 | GO Biological Processes | B cell proliferation | 6 | 2.81690140845 | -3.61832520844 | -0.611160024697 |
| GO:0002250 | GO Biological Processes | adaptive immune response | 14 | 6.57276995305 | -3.46910666327 | -0.548961588002 |
| GO:0006874 | GO Biological Processes | cellular calcium ion homeostasis | 12 | 5.6338028169 | -3.26191597186 | -0.376533002848 |
| GO:0033194 | GO Biological Processes | response to hydroperoxide | 3 | 1.40845070423 | -3.21360561262 | -0.374725808904 |
| GO:0002437 | GO Biological Processes | inflammatory response to antigenic stimulus | 4 | 1.8779342723 | -3.18372043205 | -0.374725808904 |
| R-HSA-2142691 | Reactome Gene Sets | Synthesis of Leukotrienes (LT) and Eoxins (EX) | 3 | 1.40845070423 | -3.01051717504 | -0.312220849391 |
| R-HSA-198933 | Reactome Gene Sets | Immunoregulatory interactions between a Lymphoid and a non-Lymphoid cell | 6 | 2.81690140845 | -2.79066270168 | -0.214582254297 |
| GO:2000378 | GO Biological Processes | negative regulation of reactive oxygen species metabolic process | 4 | 1.8779342723 | -2.74135586363 | -0.182321523598 |
| GO:0001975 | GO Biological Processes | response to amphetamine | 3 | 1.40845070423 | -2.73448981357 | -0.182321523598 |
| R-HSA-202733 | Reactome Gene Sets | Cell surface interactions at the vascular wall | 6 | 2.81690140845 | -2.69283938593 | -0.149423705059 |
| GO:0002573 | GO Biological Processes | myeloid leukocyte differentiation | 7 | 3.28638497653 | -2.64686760896 | -0.124666773413 |
| GO:0030073 | GO Biological Processes | insulin secretion | 7 | 3.28638497653 | -2.49875826003 | -0.0244767457477 |
| GO:0046717 | GO Biological Processes | acid secretion | 5 | 2.34741784038 | -2.32197243617 | 0.0 |
| GO:0090311 | GO Biological Processes | regulation of protein deacetylation | 3 | 1.40845070423 | -2.25941160685 | 0.0 |
| R-HSA-2129379 | Reactome Gene Sets | Molecules associated with elastic fibres | 3 | 1.40845070423 | -2.25941160685 | 0.0 |
| GO:0034644 | GO Biological Processes | cellular response to UV | 4 | 1.8779342723 | -2.2326691384 | 0.0 |

To further capture the relationship among terms, a subset of enriched terms were selected and rendered as a network plot, where terms with similarity > 0.3 are connected by edges. Currently we select the terms with the best p-values from each of the 20 clusters, with the constraint that there are no more than 15 terms per cluster and no more than 250 terms in total. The network is visualized with Cytoscape^5^, where each node represented an enriched term and colored by its cluster ID (Figure 2.a) and then by p-value (Figure 2.b). These networks can be visualized interactively in Cytoscape with the .cys files (contained in the Zip package, which also contains publication-quality pdf version.) or within browser by clicking on the web icon. For clarity, term labels are only shown for one term per cluster, so it is recommended to use Cytoscape or browser to visualize the network in order to inspect all node labels. One can also export the network into pdf format within Cytoscape and then edit labels with Adobe Illustrator for publication purposes. To switch off all labels, delete the "Label" mapping under the "Style" tab within Cytoscape and then export network view.

Figure 2. Network of enriched terms: (a) colored by cluster ID, nodes share the same cluster are typically close to each other; (b) colored by p-value, terms containing more genes tend to have a more significant p-value.

|  |  |
| --- | --- |
|  |  |
|  |  |

### Protein-protein Interaction Enrichment Analysis

For each given gene list, protein-protein interaction enrichment analysis was carried out with the following databases: BioGrid^6^, InWeb\_IM^7^, OmniPath^8^. The resultant network contains the subset of proteins that form physical interactions with at least another list member. If the network contains 3 to 500 proteins, Molecular Complex Detection (MCODE) algorithm^9^ was further applied to identify densely connected network components. The MCODE networks identified for individual gene lists were pooled and shown in Figure 3.

Pathway and process enrichment analysis was applied to each MCODE components independently and the three best-scoring (by p-value) terms were retained as the functional description of the corresponding components, shown in a table underneath each network plot within Figure 3.

Figure 3. Protein-protein interaction network and MCODE components identified in the gene lists.

| GO | Description | Log10(P) |
| --- | --- | --- |
| R-HSA-500792 | GPCR ligand binding | -8.0 |
| R-HSA-373076 | Class A/1 (Rhodopsin-like receptors) | -7.9 |
| GO:0006874 | cellular calcium ion homeostasis | -7.1 |

| Color | MCODE | GO | Description | Log10(P) |
| --- | --- | --- | --- | --- |
|  | hits\_SUB2\_MCODE\_1 | GO:0007200 | phospholipase C-activating G-protein coupled receptor signaling pathway | -6.9 |
|  | hits\_SUB2\_MCODE\_1 | R-HSA-416476 | G alpha (q) signalling events | -6.1 |
|  | hits\_SUB2\_MCODE\_1 | GO:0007204 | positive regulation of cytosolic calcium ion concentration | -5.7 |
|  | hits\_SUB2\_MCODE\_2 | R-HSA-373076 | Class A/1 (Rhodopsin-like receptors) | -5.5 |
|  | hits\_SUB2\_MCODE\_2 | R-HSA-418594 | G alpha (i) signalling events | -5.3 |
|  | hits\_SUB2\_MCODE\_2 | R-HSA-500792 | GPCR ligand binding | -5.1 |

### Reference

1. Tripathi S. et al., Meta- and Orthogonal Integration of Influenza "OMICs" Data Defines a Role for UBR4 in Virus Budding. Cell Host & Microbe (2015) 18:723-735.
2. Zar, J.H. Biostatistical Analysis 1999 4th edn., NJ Prentice Hall, pp. 523
3. Hochberg Y., Benjamini Y. More powerful procedures for multiple significance testing. Statistics in Medicine (1990) 9:811-818.
4. Cohen, J. A coefficient of agreement for nominal scales. Educ. Psychol. Meas. (1960) 20:27-46.
5. Shannon P. et al., Cytoscape: a software environment for integrated models of biomolecular interaction networks. Genome Res (2003) 11:2498-2504.
6. Stark C. et al. BioGRID: a general repository for interaction datasets. Nucleic Acids Res. (2006) 34:D535-539.
7. Li T. et al. A scored human protein-protein interaction network to catalyze genomic interpretation. Nat. Methods. (2017) 14:61-64.
8. Turei D. et al. A scored human protein-protein interaction network to catalyze genomic interpretation. Nat. Methods. (2016) 13:966-967.
9. Bader, G.D. et al. An automated method for finding molecular complexes in large protein interaction networks. BMC bioinformatics (2003) 4:2.
